# Investigating physical and mechanical properties of nest soils used by mud dauber wasps from a geotechnical engineering perspective

**DOI:** 10.1038/s41598-022-06162-2

**Published:** 2022-02-09

**Authors:** Joon S. Park, Noura S. Saleh, Hai Lin, Hussein Alqrinawi, Nathan P. Lord

**Affiliations:** 1 Department of Civil and Environmental Engineering, Louisiana State University, Baton Rouge, LA 70803 USA; 2 Department of Entomology, Louisiana State University, Baton Rouge, LA 70803 USA

**Keywords:** Civil engineering, Entomology

## Abstract

The quality of nest soils has significant effects on reproductive success in mud dauber species. This study investigated the physical and mechanical properties of the nest soils used by mud daubers from a geotechnical engineering perspective. One hundred thirty-one nests of black and yellow mud daubers were collected from five locations in the south of Louisiana. Moisture and organic contents, densities, void ratios, plasticity, grain size distributions, soil classifications, and penetration resistances of the nest soils were measured. Also, the performance of mud daubers’ nest-compaction method (i.e., repetitive tapping produced by the front legs and mandibles) was evaluated by comparing the densities and penetration resistances between mud dauber nests and Proctor compacted nest soil samples. Scanning electron microscopy, energy-dispersive X-ray spectroscopy, and X-ray diffraction were used to measure the morphology, elemental composition, and mineralogy of the nest soils. Mud dauber nests were made of hard and very stiff well-graded silty soils. The high strengths and high densities of mud dauber nests were attributed to repetitive tapping (similar to vibratory compaction in geotechnical engineering) used by mud daubers for nest construction, high capillary cohesion in the nest soils, well-graded soil grain size distribution, and clay minerals serving as cementing agents in the nest soils.

## Introduction

Sceliphron mud daubers, such as the black and yellow mud dauber (*S. caementarium*), are common solitary sphecid wasps in the United States^1,2^. Mud dauber wasps are solitary (i.e., do not live in colonies) and do not have a caste system (e.g., workers and queens)^3^. Mud daubers build soil nests for their offspring using local soils^4^. Mud dauber nests are often built on man-made structures, such as under roofs and eaves, in attics, under bridges, and on walls of buildings^1,2^. Black and yellow mud dauber nests have rectangular-like shapes and are typically composed of several tubular brood cells arranged side by side, where a female mud dauber stores prey (i.e., spiders) and lays her eggs^1^.

**Figure 1:**
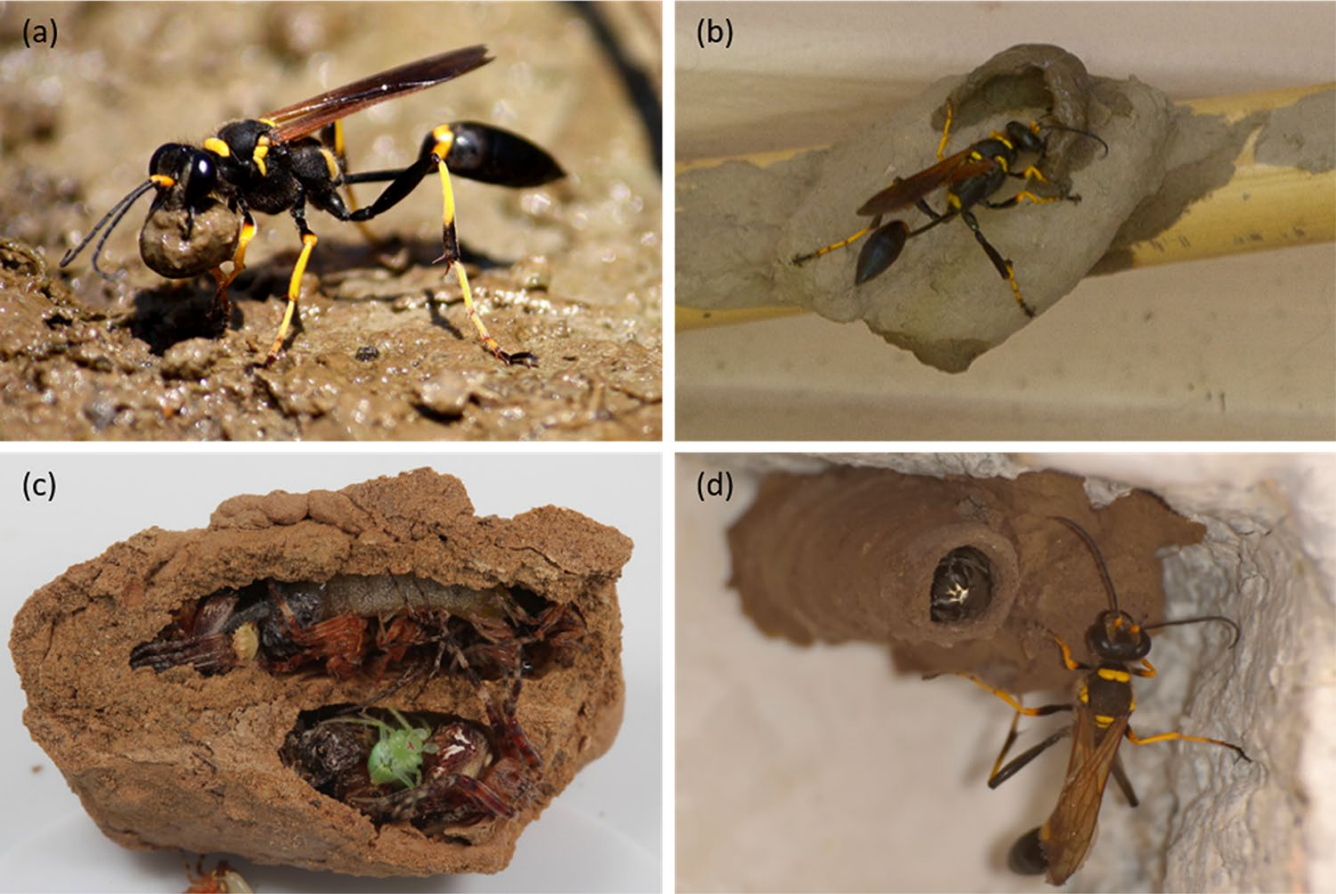
Nest construction process of black and yellow mud dauber: (**a**) a mud dauber collects and forms soil into a soil ball and (**b**) plasters the soil ball on the nest; (**c**) nest cells are provisioned with spiders; and (**d**) a mud dauber closes the opening of the cell using collected soil. (**a**): photo courtesy of an author named Hglu1 via https://en.wikipedia.org/wiki/Sceliphron_caementarium#/media/File:20100710.MudDauber-SceliphronCaementarium.Hannibal.jpg. The photo is licensed under a Creative Commons Attribution-ShareAlike 3.0 Unported license. The license terms can be found on the following link: https://creativecommons.org/licenses/by-sa/3.0/deed.en. (**b**), (**c**), and (**d**): photo courtesy of authors named mnwild, Patrick Coin, and Ricardo Arredondo T. via https://www.inaturalist.org/observations/39302921, https://www.inaturalist.org/observations/8419859, and https://www.inaturalist.org/observations/10717949. These photos are licensed under a Creative Commons Attribution-NonCommercial 4.0 International license. The license terms can be found on the following link: https://creativecommons.org/licenses/by-nc/4.0/.

Mud daubers emerge from last year's nests in late spring to mid-summer^5^. After mating, females begin to construct their soil nests near a reliable water source, soil, and abundant spiders^5^. Female mud dauber collects soil near the selected nest location by digging soil with its vibrating mandibles controlled by the wing muscles^6–8^. She then forms the soil into a ball and carries it back to the nest by holding it with her front legs and mandibles (Fig. 1a)^9–11^. Once the female mud dauber arrives at the nest, she lays and plasters the soil ball on the nest by rolling it into a cell wall layer (Fig. 1b)^12^. At the same time, the plastered soil layer is compacted by the repetitive tapping produced by the front legs and vibrating mandibles, creating an intense vibration buzzing noise^7,13^. This repetitive tapping on soil plays a similar role as the vibratory compaction commonly used to compact soil in geotechnical engineering^14^. The cells are built in a concentric way around the lumen^15^. Each cell is constructed with about 30 to 40 soil ball collecting trips, requiring several hours to a full day’s labor^3,11,16,17^. Once the cell is completed, a period of inactivity (about 1 h to 2 days) is given to allow the new cell to dry (i.e., drying induced soil hardening)^18^. The completed cell is then provisioned with sting-paralyzed spiders (up to 25 spiders) (Fig. 1c)^3,19^. Only one egg is laid on the abdomen of one of the spiders in each brood cell^12^. The female then gathers more soil to cap the opening of the cell and begins building the next cell (Fig. 1d)^12^. After constructing multiple cells, a final thick soil layer is sometimes constructed to cover the cells, which is likely to offer additional protection against predators and prevent the nests from disintegrating under rainwater^3,15^.

The quality of mud dauber nests has significant effects on reproductive success of mud daubers^4,12^. Although their nest construction, foraging behavior, and load-lifting capability have been extensively studied^6,9,10,15,20^, little is known about the physical and mechanical properties of mud dauber nest soils, which controls their nest quality.

Similarly, soil properties play a critical role in the quality of nests of other animals that build their nests with soil. Characterizations of soil types and properties were conducted on nest soils used by termites and hirundine species^21,22^. It has been shown that these animals use specific soil types, cementation additives, and unique construction techniques to improve soil properties for nest construction. For example, termites fabricate boluses by cementing soil particles with their salivary secretions as building bricks for their termite mound construction^21,23^. Also, strong capillary actions developed by clay particles significantly enhance the termite mound strength^24^. Due to the platy shape of clay particles, the water is sandwiched between the platy clay particles, developing high capillary attraction (i.e., matric suction or capillary cohesion)^25^. Similarly, hirundine species use clay minerals and saliva as gluing agents to cohere with collected sandy and silty soils for nest construction^22,26^. Specific data on the soil type of Sceliphron mud dauber nests have been reported before by Polidori et al.^15^, who reported that the nest soil was composed of about 55% sand and 45% silt and was independent of geographical location. They also reported that salivary secretions were not used for cementing the nest soil based on the low amount of organic matter measured in the nest soil. However, other physical and mechanical properties of mud dauber nest soil (e.g., strength, density, moisture content, etc.) have not been systematically investigated.

In this paper, we attempted for the first time to investigate the physical properties (e.g., moisture and organic contents, density, void ratio, plasticity, grain size distribution, and soil classification) and strength of the nest soils used by the black and yellow mud daubers from a geotechnical engineering perspective. The densities and strengths of the mud dauber soil nests were also compared to those of the nest soil samples compacted by Proctor tests to evaluate the performance of mud daubers’ repetitive tapping (i.e., vibratory compaction) on soil compaction. Finally, the factors that contribute to increasing the strength and density of mud dauber soil nests were discussed.

## Methods

A series of tests were performed to investigate the physical and mechanical properties of the nest soils. The intact nests were first subjected to penetrometer tests to measure penetration resistances. Several physical properties were then measured using the nest samples after penetrometer tests, including moisture contents, organic contents, dry densities, and specific gravities. After these tests, the remaining nest samples were smashed using a mortar and pestle and used for grain size analysis and soil classification, which was performed based on the unified soil classification system (USCS) and the revised soil classification system (RSCS)^27–29^. To explore the morphology, elemental composition, and mineralogy of the nest soils, scanning electron microscopy (SEM), energy-dispersive X-ray spectroscopy (EDS), and X-ray diffraction (XRD) analysis were also conducted. Furthermore, to evaluate the performance of mud daubers’ vibratory compaction on soil compaction, laboratory Proctor compaction tests were performed to compact the smashed nest soils. The measured dry densities and penetration resistances from the Proctor compaction tests were compared to those of the mud dauber nests.

### Nest samples

One hundred thirty-one nests of black and yellow mud daubers were collected from five locations in the south of Louisiana (see Supplementary Fig. S1). Ninety-four samples were collected at Location A near the Jean Lafitte National Park, Jefferson Parish. Twelve, ten, and nine nests were collected at Locations B, C, and D, respectively, on the campus of Louisiana State University at Baton Rouge. Six nests were collected at Location E, located near the city of Lafayette. The nests were gently detached from the walls and eaves using a putty knife. The collected nest samples were then stored in a sealable plastic bag to minimize moisture loss. The nests are amorphous with rectangular-like shapes (see Supplementary Fig. S2a) or trapezoidal-like shapes (see Supplementary Figs. S2b and c). Cylindrical brood cells in a nest sample are arranged side by side and stacked in a pyramidal manner (see Supplementary Fig. S2c). There are up to 11 brood cells in a nest sample. The dimensions of the collected nest samples ranged from 2 to 12 cm in length, 3 to 7 cm in width, and 1 to 4 cm in height. The nest soil colors include light brown, brown, light gray, gray, and white.

### Penetrometer tests

Due to the amorphous nest shape and tubular brood cells in the nests, it is unable to perform conventional soil strength tests (e.g., unconfined compression and triaxial tests). Therefore, this study used an electrical penetrometer device (GY-4 Digital Fruit Firmness Tester) to measure the penetration resistances of mud dauber nests. The penetrometer has a tip with a diameter of 3.5 mm that is connected to a load cell with a capacity of 500 N. During penetrometer tests, the penetrometer tip was slowly pushed into the nest samples until the penetration reached 6 mm according to the testing procedure of the pocket penetrometer (commonly used in geotechnical engineering and soil investigation^30^). The maximum loads applied to the samples were recorded and calibrated to the pocket penetrometer readings. These converted pocket penetrometer readings were defined as the penetration resistances of the nests. Spiders and wasp pupas were removed from the brood cells after penetrometer tests. The penetrometer tests were continued to run on the broken nest samples until the thickness of the nest samples is less than 20 mm to minimize boundary effects. The broken samples after penetrometer tests were then used to measure physical properties. A total of 711 penetrometer tests were conducted, including 614, 14, 23, 25, and 35 tests for Locations A, B, C, D, and E, respectively (see Supplementary Table S1). The number of penetrometer tests of each location depends on the number and size of the available nest samples.

### Moisture contents, organic contents, and dry densities

After penetrometer tests, nest samples were used for measuring moisture (131 tests) and organic (131 tests) contents (see Supplementary Table S2). Moisture content was calculated by dividing the sample mass difference before and after oven-drying (105 °C for 24 h) by the dry sample mass according to ASTM D2216^31^. The samples were then subjected to heating in the muffle furnace at 550 °C for 2 h to burn off organic matters. The mass of the organic matter was determined as the mass difference before and after muffle furnace heating. The organic content was then calculated by dividing the mass of organic matter by the dry sample mass without organic matter. Dry density measurements were also conducted on intact nest samples coated by wax following ASTM D7263^32^. Dry density was calculated using Eq. (1),

1 $$\rho_{d} = \frac{{M_{t} }}{{\left[ {\left( {M_{c} - M_{sub} } \right)/\rho_{w} } \right) - \left( {M_{c} - M_{t} /\rho_{\rho } } \right)]}} \times \frac{1}{1 + w} = \frac{{\rho_{m} }}{1 + w}$$

where $M_{t}$ = mass of the nest sample; $M_{c}$ = mass of the wax-coated sample; $M_{sub}$ = mass of the submerged wax-coated sample; $\rho_{w}$ = density of water; $\rho_{\rho }$ = density of wax; $\rho_{m}$ = moist density of the nest sample; $\rho_{d}$ = dry density of the nest sample; and $w$ is the moisture content of the nest sample. Additional samples from different sample collection locations were used for measuring specific gravities in accordance with ASTM D854^33^. Since the measured specific gravities from five nest collection locations showed little variation (from 2.59 to 2.62), the average specific gravity (2.6) was used. Based on the measured dry densities and average specific gravity, void ratios (the ratio between the volume of voids and the volume of solids) of the nest soils were calculated using Eq. 2,

2 $$e = \frac{{G_{s} }}{{\rho_{d} }} - 1$$

where $\rho_{d}$ = dry density of the nest sample; $G_{s}$ = average specific gravity; and $e$ = void ratio of the nest soil sample. The nest soils used in the tests of moisture and organic contents and dry densities were not used for the following tests.

### Grain size distributions and soil classifications

The grain size distributions were measured for the nests from locations A, B, C, and D except for location E due to the limited availability of nest samples. An automated hydrometer (PARIO, Meter Group), which can measure soil particle size ranging from 2 to 63 μm, was used to measure the grain size distributions of nest soils. PARIO estimates grain size distribution based on Stoke’s law, which measures the integral suspension pressure changed by particle sedimentation with time. Fifty grams of nest soils from each location (A, B, C, and D) were used for grain size distribution measurements. To remove the effect of organic matters on the grain size distribution, organic matters in nest soils were removed by mixing the nest soil samples with 15% hydrogen peroxide for 12 h followed by heating at 80 °C for 4 h. The nest soils were then collected using a centrifuge. Next, deflocculating solution (100 mL of 5% sodium hexametaphosphate) and distilled water (250 mL) were added to the nest soil samples^34^. The mixture was placed on a reciprocating shaker for 12 h to disperse soil particles. The mixture was then transferred to the 1L cylinder. Distilled water was added to fill the rest of the cylinder space. The 1L cylinder was shaken by repeatedly turning it upside down and back for 30 cycles in 1 min. The PARIO hydrometer was then lowered into the cylinder to start the hydrometer tests. After the PARIO hydrometer tests, soil samples were wet sieved with No. 270 sieve (opening size = 53 μm). The fraction retained on the sieve was used to perform sieve analysis. Sand fractions corresponding to the size ranges of 53 to 250 μm, 250 to 500 μm, and 500 to 2000 μm were obtained from the sieve analysis. The results of PARIO hydrometer tests were then combined with the measurements of the sieve analysis to produce grain size distributions of the nest soils^35^. The grain size distributions were then used to classify the nest soils based on the unified soil classification system (USCS). The nest soils used in the grain size distribution measurements were discarded and not used for the following compaction tests.

The revised soil classification system (RSCS) was also used to classify the nest soils and measure the plasticity according to Park and Santamarina^29^. Compared to the USCS, the RSCS can better capture the dominant soil particle sizes (i.e., coarse or fine soils) that control the mechanical and hydraulic properties. The required input parameters for producing the classification charts include sand fraction percentage, *F*_*s*_ (0.075 mm < particle size < 4 mm), fine fraction percentage, *F*_*F*_ (particle size < 0.075 mm), coefficient of uniformity of sand fraction, ${C}_{u}$, and sand grain roundness, *R* (see Supplementary Table S3). The first three parameters can be obtained from the grain size distributions. The roundness was estimated by visually comparing grain shapes observed from an optical microscope against particle shape charts in Krumbein and Sloss^36^. Triangular soil classification charts were developed for the nest soil samples using the Excel macro provided by Park and Santamarina^29^. The triangular charts were divided into several zones with different RSCS classifications. The soil samples were then classified based on their locations in the triangular charts. The RSCS classification was determined using a two-name nomenclature. The first letters of the nomenclature identify the component that controls mechanical properties, and the following letter in parenthesis identifies the component that controls hydraulic properties^29^. In addition, fall cone tests^37^ were conducted for soil samples passing sieve No. 200 (opening size = 0.075 mm) at locations A and D to measure liquid limits^29^. The liquid limits of soil samples from locations B, C, and E were not measured due to their limited availability.

### Compaction tests

To evaluate the compaction performance of mud daubers’ vibratory compaction, the measured dry densities and penetration resistances of the intact nest samples were compared to those of Proctor compacted nest soil samples. Standard and modified Proctor compaction tests were conducted in accordance with ASTM D698^38^ and D1557^39^. Due to the limited availability of nest samples from locations B, C, D, and E, standard and modified compaction tests were only performed on the nest soils from location A. For standard Proctor tests, loose nest soils were compacted in a mold with the diameter of 101.6 mm and height of 116.4 mm by dropping a 24.5 N rammer from the height of 205 mm for 75 times (25 times for each of the three layers), producing a compactive effort of 600 kJ/m^3^. For modified Proctor tests, the same mold was used, while a 44.5 N rammer was dropped from a height of 457.2 mm 125 times (25 times for each of the five layers), producing a compactive effort of 2,700 kJ/m^3^. In each Proctor test, five samples with different moisture contents were subjected to the same compaction effort to produce a relationship between the dry densities and moisture contents of the nest soils. The dry densities achieved in the standard and modified Proctor tests were compared to the dry densities of nest samples to assess the performance of mud dauber’s vibratory compaction on soil compaction. After Proctor compaction tests, penetrometer tests were performed on the compacted soil samples with maximum dry densities to compare their penetration resistances with the mud dauber nest samples. The compacted soil samples with the maximum dry densities were ejected out of the mold and dried in an oven at 30 °C until the moisture contents reached approximately 2% (similar to the mean moisture content of the mud dauber nest samples). This drying process was assumed as an equivalent process of mud dauber nests subjected to air drying. The compacted soil samples were then broken into smaller specimens with similar thickness as the intact mud dauber nests. These smaller soil specimens were then subjected to penetrometer tests following the same procedure described before. A total of 444 and 379 penetrometer tests were conducted for the soil specimens subjected to standard and modified Proctor tests, respectively (see Supplementary Table S4).

### SEM, EDS, and XRD analysis

Raw nest samples were saved and dried at 105 °C for at least 24 h for SEM imaging and EDS analysis. The Quanta 3D Dual Beam SEM was used for investigating the morphology and soil structure of the mud dauber nests. The EDS was integral to the SEM and was used to analyze the elemental compositions of the nests. Morphology of the samples was observed under SEM using Everhart–Thornley detector at an acceleration voltage of 5 kV and magnification of 500 to 1500x. XRD analysis was also performed to investigate the mineralogy of the nest soils. To prepare XRD samples, raw nest samples from each location were smashed using a mortar and pestle and then dried at 105 °C for at least 24 h. The XRD patterns were obtained in a PANalytical Empyrean X-ray Diffractometer using Cu-Kα radiation (λ = 1.54 Å) with excitation energy of 40 mA and 45 kV, a step size of 0.026°, and diffraction angles (2θ) of 5° to 80°.

## Results

### Penetration resistances

**Figure 2:**
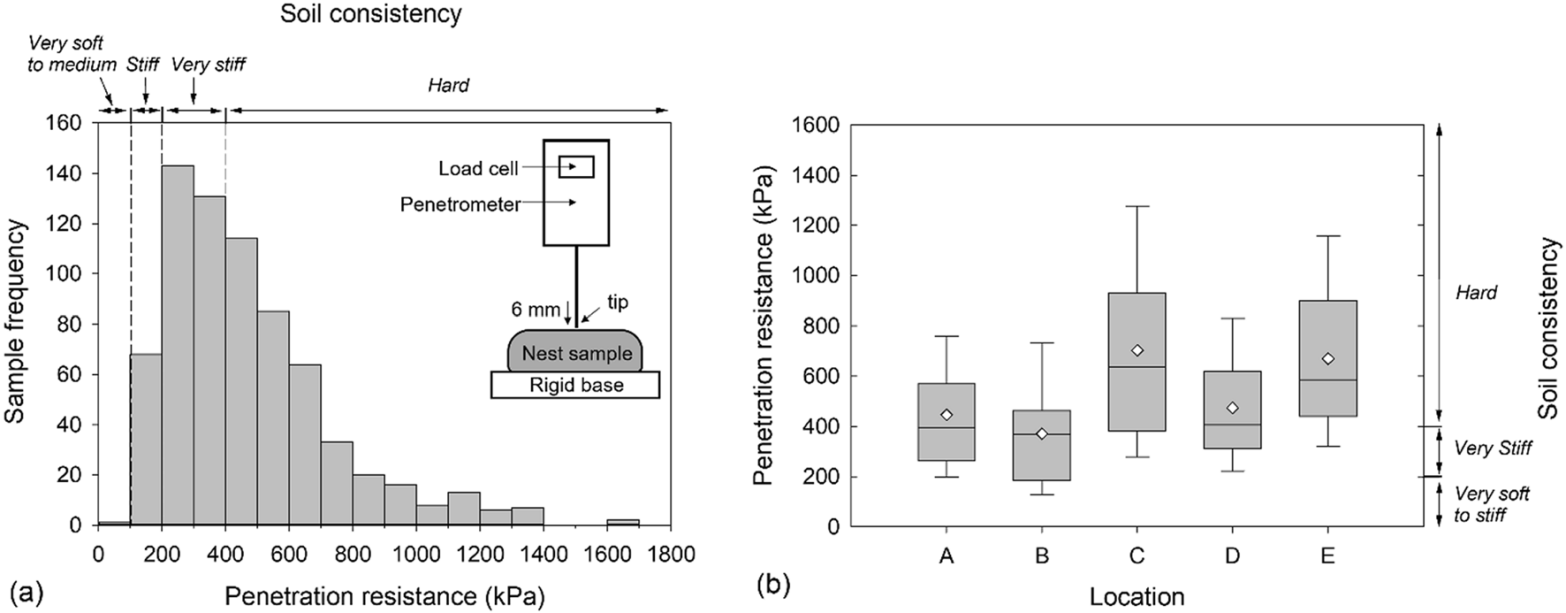
Penetrometer tests: (**a**) the histogram of penetration resistances of collected mud dauber nest samples and (**b**) box plot of penetration resistances by sample collection locations. The terms to describe soil consistency include: hard (greater than 400 kPa), very stiff (between 200 and 400 kPa), stiff (between 100 and 200 kPa), medium (between 50 and 100 kPa), soft (between 25 and 50 kPa), and very soft (less than 25 kPa).

Penetration resistances of mud dauber nest samples are presented in Fig. 2a. The statistical data of penetration resistances are summarized in Supplementary Table S1. The mean of penetration resistances is 466 kPa and the median is 408 kPa. The interquartile range (IQR) of penetration resistances, which corresponds to the range between the first quartile (Q1 = 25% of the data is smaller than Q1) and the third quartile (Q3 = 75% of the data is smaller than Q3), is between 268 and 585 kPa. The median is less than the mean, indicating the distribution is right skewed as shown in Fig. 2a. According to Holtz and Kovacs^30^, the penetration resistance of a soil sample is equal to its unconfined compressive strength, which can be used to determine the soil consistency. Soil consistency corresponds to soil density and is evaluated by noting the ease with which the soil can be penetrated by one’s fingers^30^. The terms used to describe soil consistency include very soft, soft, medium, stiff (or firm), and hard, which corresponds to different ranges of unconfined compressive strengths as shown in Fig. 2a. When evaluating the consistencies of the nest soils based on the measured penetration resistances (Fig. 2a), 51% of samples are hard, 39% of samples are very stiff, 10% of samples are stiff, and very limited samples are medium. This indicates that mud daubers produced strong and dense soil nests.

The penetration resistances by nest collection locations are plotted in Fig. 2b. The minimum, first quartile (Q1), median, third quartile (Q3), and maximum are plotted as horizontal lines. The mean is plotted as a diamond symbol. These statistical data are also summarized in Supplementary Table S1 by collection locations. The distributions of penetration resistances at locations A, B, and D are similar. However, there are larger ranges of penetration resistances and higher mean and median values at locations C and E. The higher penetration resistances of location C may be due to the higher soil densities at location C, which will be discussed in the next section. It is unclear why location E has higher penetration resistances than locations A, B, and D.

### Moisture contents, organic contents, dry densities, and void ratios

**Figure 3:**
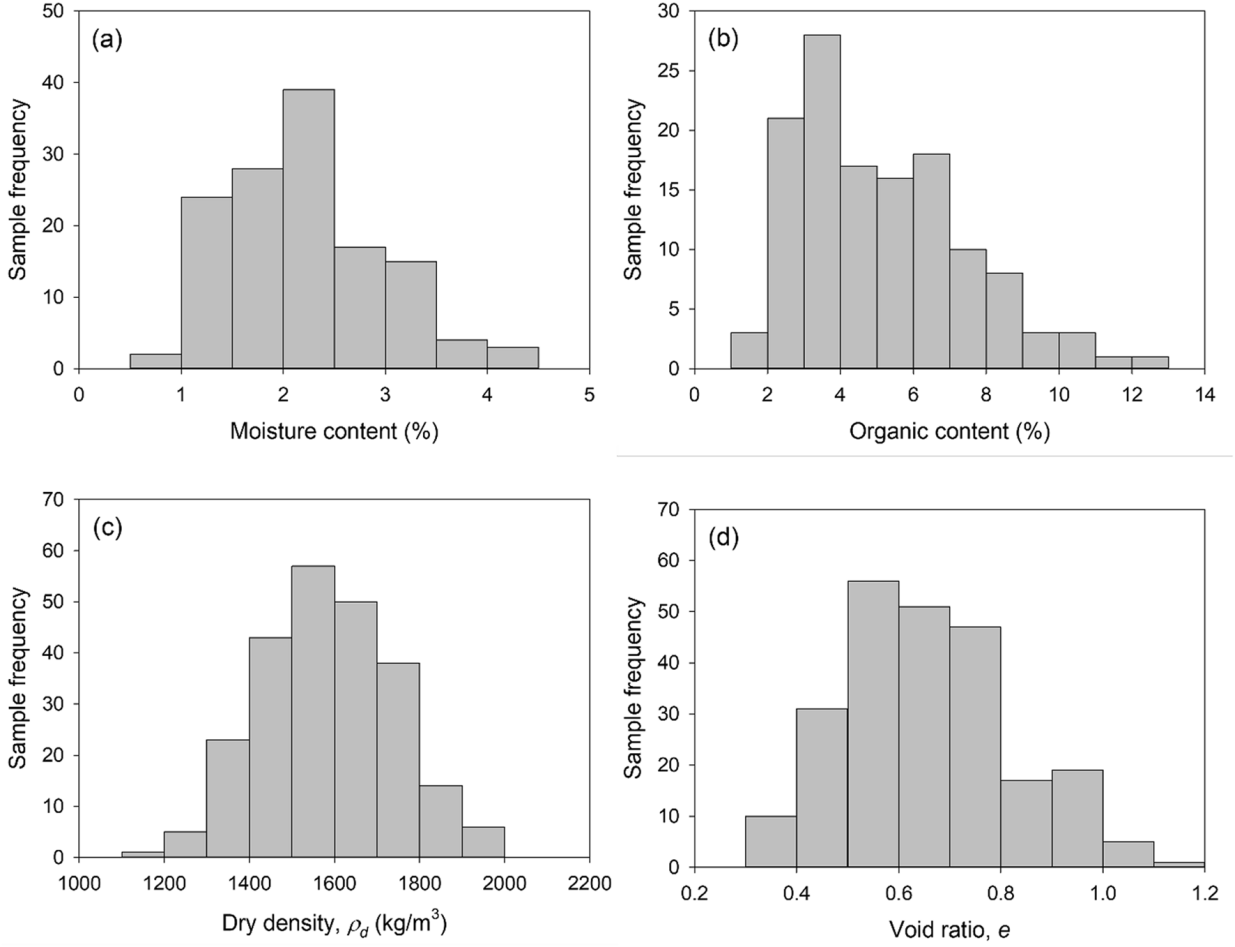
Physical properties of nest samples: (**a**) moisture content, (**b**) organic content, (**c**) dry density, and (**d**) void ratio.

The distributions of moisture contents, organic contents, dry densities, and void ratios are presented in Fig. 3. The corresponding statistical data are summarized in Supplementary Table S2. As shown in Fig. 3a, moisture contents are distributed from 0.65 to 4.12% with IQR ranging from 1.71 to 2.72%. Their mean and median are 2.22% and 2.17%, respectively. Since mud dauber nests are located at the sheltered locations (e.g., under roofs and eaves, in attics, and under bridges) that protect the nests from rainwater, this low moisture content range is equilibrated with the atmospheric humidity level and temperature at the nest locations. The mean and median of the organic contents are 5.16% and 4.78%, respectively. The IQR of the organic contents ranged between 3.33 and 6.70%. The organic contents of the nest soils are highly affected by the organic matter contents in the surrounding soil near nest locations. Also, the salivary secretions of mud daubers may contribute to the measured organic contents. However, whether or not mud daubers use salivary secretions during nest construction has not been ascertained in the literature^15,40,41^, which will be further discussed in the discussion section. The mean and median of dry densities, 1587 and 1588 kg/m^3^, are approximately the same, implying dry densities are normally distributed. The IQR of dry densities is between 1,483 and 1699 kg/m^3^, which is as high as the dry densities achieved in the standard Proctor compaction tests (will be discussed in compaction tests). The high dry densities of nest soil samples can be attributed to the grain size distribution of the soil collected by mud daubers (will be discussed in grain size distributions and soil classifications) and the vibratory compaction technique (i.e., repetitive tapping produced by the front legs and vibrating mandibles) used by mud daubers during nest construction. The average specific gravity of the nest soil samples from five locations was measured as 2.6, which is slightly lower than the typical specific gravity of 2.65 for inorganic soils. This low specific gravity is probably attributed to the organic matter existing in the nest soil. Based on the measured dry densities and average specific gravity, void ratios of nest soils can be calculated using Eq. 2. The mean, median, and IQR of void ratios are 0.65, 0.64, and from 0.53 to 0.75, respectively.

**Figure 4:**
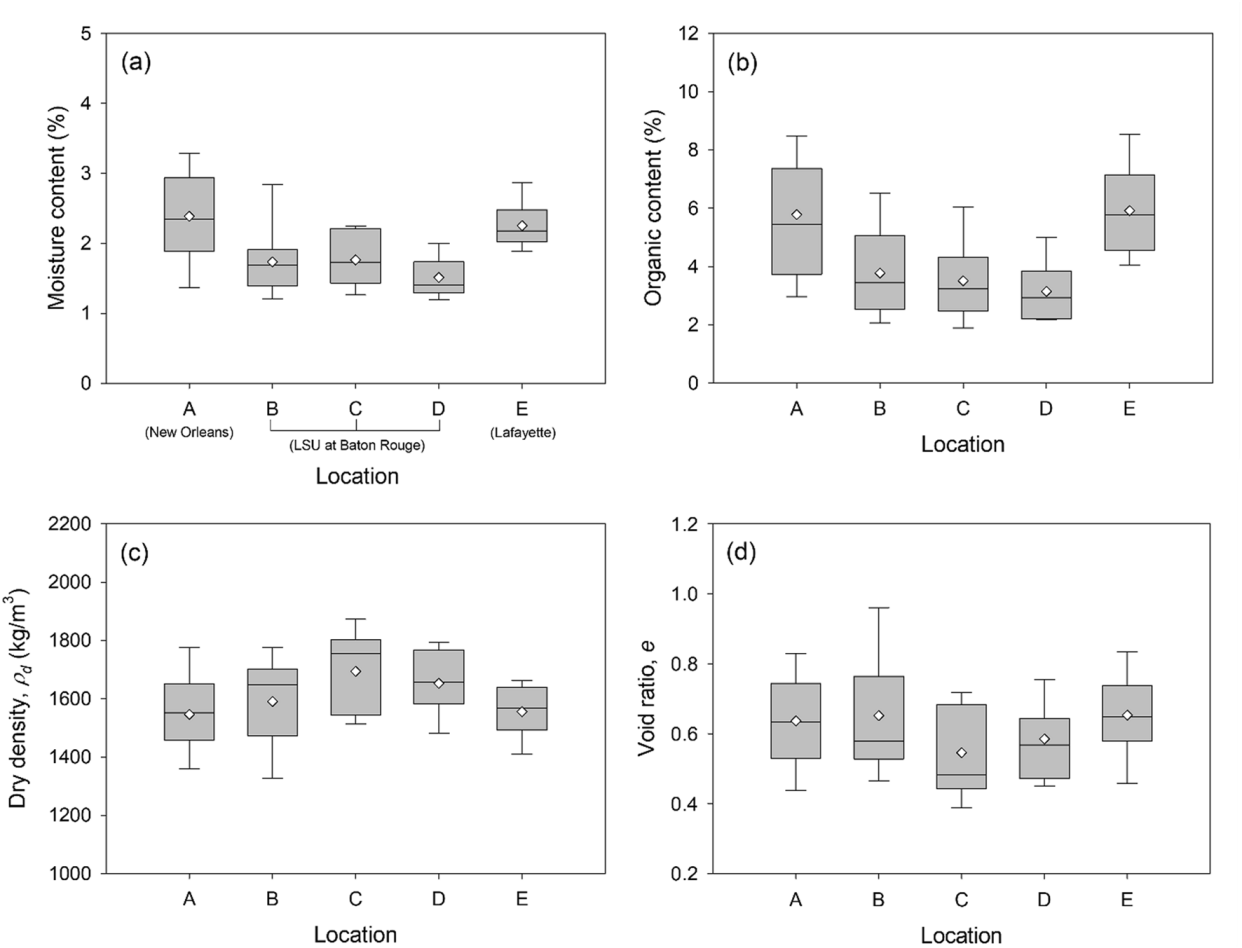
Comparisons of the physical properties of the nest samples by locations: (**a**) moisture content, (**b**) organic content, (**c**) dry density, and (**d**) void ratio. Horizontal lines extending from the boxes through the whiskers indicate the minimum and maximum values. Horizontal edges of the boxes indicate the first quartile (Q1) and the third quartile (Q3). The horizontal line in the middle of the box indicates the median. The mean is shown as the diamond symbol.

The dependence of the physical properties on sample collection locations was investigated using box plots by sample collection locations (Fig. 4). The statistical data (e.g., minimum, Q1, median, Q3, and maximum) were plotted in the same way as Fig. 2b. These statistical data are also summarized in Supplementary Table S2. As shown in Fig. 4a and b, moisture contents and organic contents are varying at different nest locations. Location A has broader distributions of moisture and organic contents than other locations. Locations B, C, and D have similar moisture and organic contents as these locations are close on the LSU campus. Location E has different ranges of moisture and organic contents with locations B, C, and D. It can be observed that the moisture and organic contents of mud dauber nests are probably controlled by the climate (e.g., humidity level and temperature) and soil conditions (e.g., organic contents) at different nest locations. However, as shown in Fig. 4c and d, the variations of dry densities and void ratios are similar between different locations, which indicates that the dry densities and void ratios may be less dependent on nest locations. The dry densities and void ratios may be controlled by the soil types and vibratory compaction used by mud daubers during nest construction.

### Grain size distributions and soil classifications

**Figure 5:**
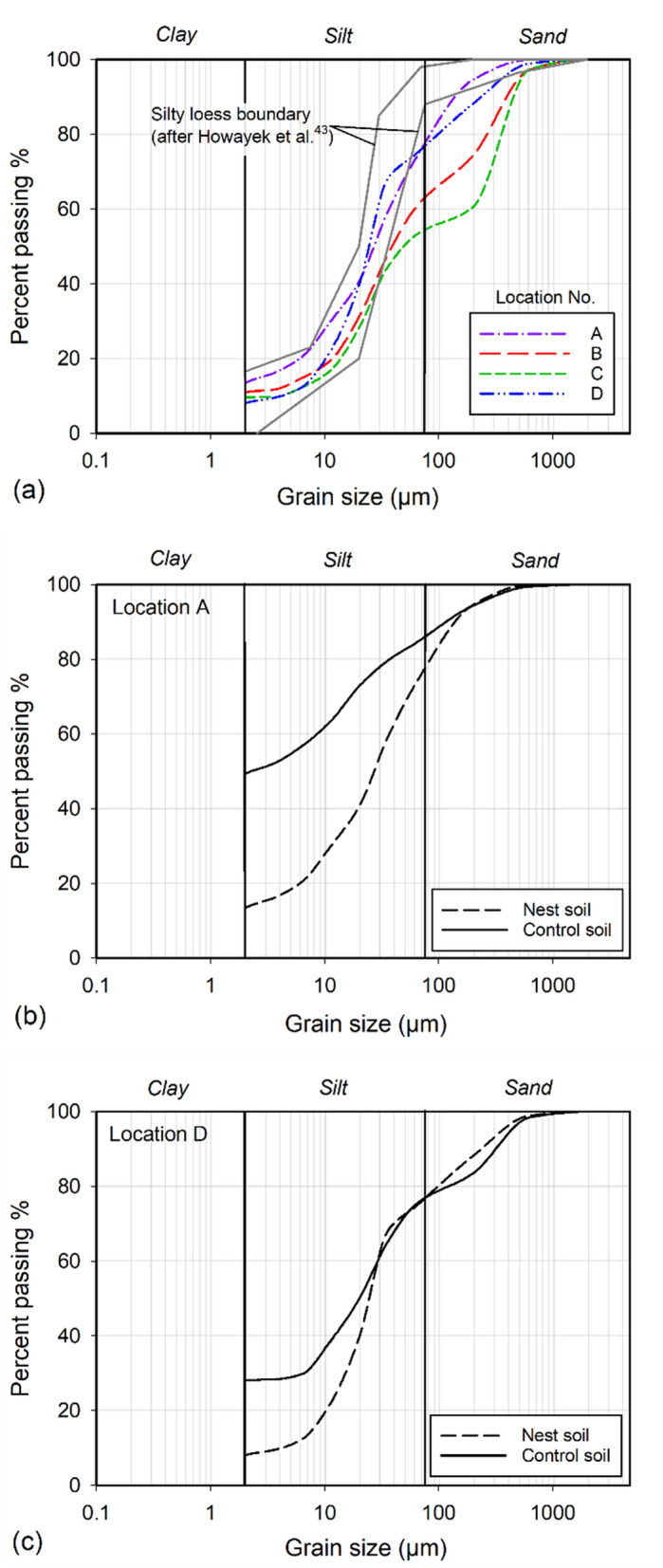
Grain size distributions of (**a**) the nest soil samples from different locations, (**b**) the nest soil sample compared to the control soil at location A, and (**c**) the nest soil sample compared to the control soil at location D.

The grain size distributions of the nest soils from locations A, B, C, and D are presented in Fig. 5a. The grain size distributions show that the nest soils are composed of silt fraction ranging from 44.9% to 68.8%, sand fraction ranging from 22.5% to 45.5%, and clay fraction ranging from 8% to 13.4%. The nest soil of location A consists of 22.5% sand, 64.1% silt, and 13.4% clay, which is similar to location D where the nest soil is composed of 23.2% sand, 68.8% silt, and 8% clay. The liquid limits of the nest soils (passing sieve No. 200) from locations A and D were measured as 46.1% and 33.8%, respectively. These liquid limit measurements demonstrate that the fines of nest soils have low plasticity^28^. The nest soils from locations A and D are classified as silt with some sand and trace of clay (ML) according to the USCS. The nest soil from location B comprises 37% sand, 52% silt, and 11% clay. And the nest soil from location C consists of 45.5% sand, 44.9% silt, and 9.6% clay. The nest soils from locations B and C are classified as sandy silt (ML) or sand and silt with trace of clay based on the USCS. The grain size distributions are generally similar between different sample collection locations, demonstrating that the grain size distributions of the soils collected by mud daubers are probably independent of nest locations. In order to compare the grain size distributions of the nest soils with the control soils (i.e., surrounding soil) near nest locations, two control soils were collected near nest locations A and D and were used for grain size distribution measurements. As shown in Fig. 5b and c, there are apparent differences of the grain size distributions between the nest soils and control soils. The mud dauber nest soil at location A has 8.5% more sand, 27.5% more silt, and 36% less clay than the control soil (Fig. 5b). The mud dauber nest soil at location D has a similar amount of sand, 20.1% more silt, and 20.2% less clay than the control soil (Fig. 5c). The comparison of the grain size distributions between nest soils and control soils indicates that mud daubers can sort and collect a specific amount, size, and type of soil particles (especially silt and clay) for building their nests. This comparison also confirms that the soil particles collected by mud daubers for nest building are independent of geographical locations, which is also confirmed by Polidori et al.^15^.

The RSCS was also used to classify the nest soils. The input parameters for RSCS are presented in Supplementary Table S3. The triangular RSCS charts of nest soils from locations A, B, C, and D are shown in Supplementary Fig. S3. All soil samples are classified as F(F), represented with the yellow circle symbols (see Supplementary Fig. S3). This classification symbol indicates that the fine fraction of the nest soils is the load-carrying component (i.e., controls mechanical properties) and controls hydraulic properties of the nest soils.

### Compaction tests

**Figure 6:**
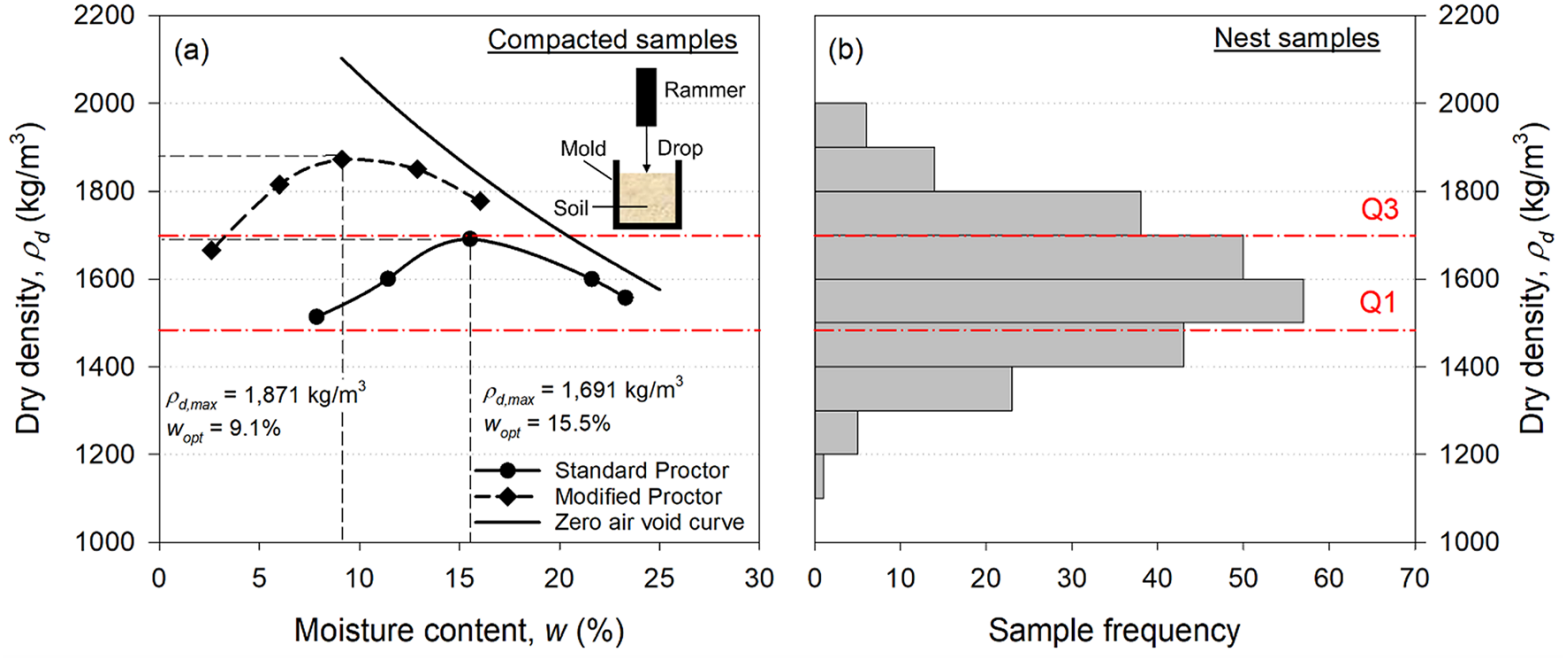
(**a**) Standard and modified Proctor compaction curves compared to (**b**) the dry density distribution of the mud dauber nest samples ( modified from Fig. 3c).

Standard and modified Proctor compaction curves are presented in Fig. 6a. The maximum dry densities ($\rho_{d, max}$) are 1,691 kg/m^3^ and 1,871 kg/m^3^ with corresponding optimal moisture contents ($w_{opt}$) of 15.5% and 9.1% for standard and modified Proctor compaction curves, respectively. The zero-air void curve is also included in Fig. 6a to show the theoretical maximum dry densities ($\rho_{zav}$) with no air voids (i.e., saturated condition) at different moisture contents^30^. The differences between $\rho_{zav}$ and $\rho_{d, max}$ at the optimal moisture contents are 162 kg/m^3^ and 230 kg/m^3^ for the standard and modified Procter compaction tests, respectively. These Proctor compaction curves were compared to the distribution of dry densities of mud dauber nests as shown in Fig. 6b to evaluate the performance of the vibratory compaction (i.e., repetitive tapping produced by the front legs and vibrating mandibles) conducted by mud daubers during nest construction. The dry density IQR of the nest samples is shown as the red dashed lines in Fig. 6a and b. It can be seen that the dry density IQR of the nest samples is comparable to the dry densities of the standard Proctor compaction curve. Also, 25% of the nest samples have dry densities above Q3 that are as high as those of the modified Proctor compaction curve. Thus, the comparison of dry densities between compaction tests and mud dauber nest samples demonstrates that the vibratory compaction conducted by mud daubers can achieve a similar dry density range as the laboratory standard Proctor compaction test with a compactive effort of 600 kJ/m^3^.

**Figure 7:**
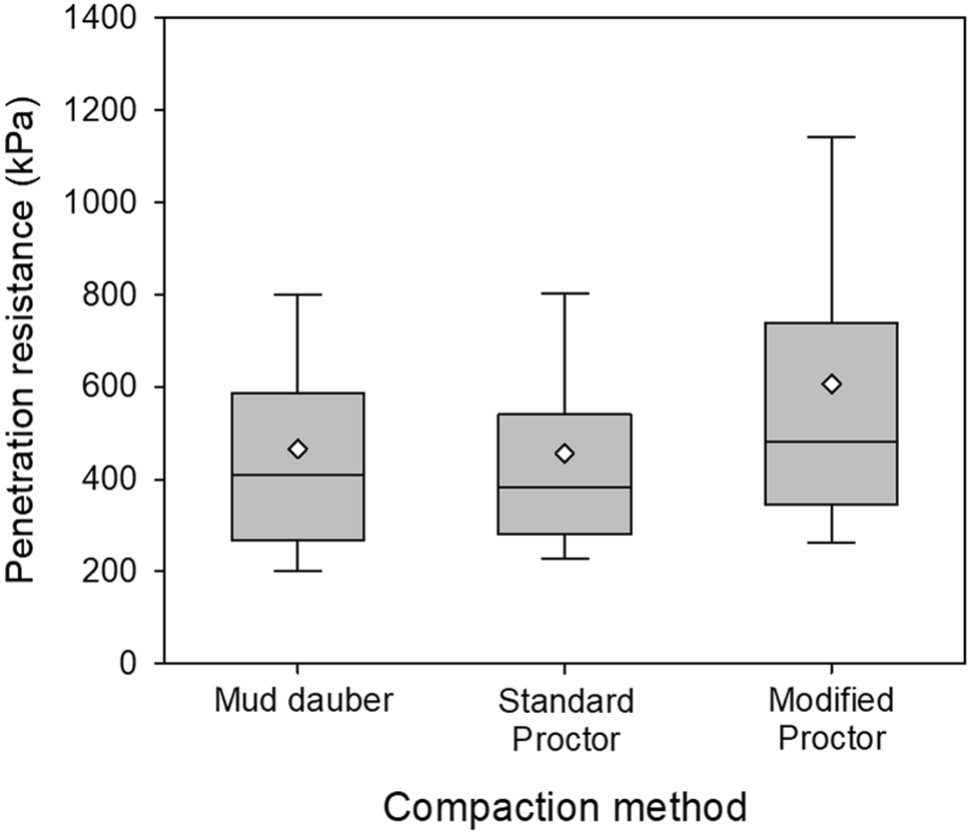
Comparison of the penetration resistances between different compaction methods.

The penetrometer tests were also performed on the compacted samples that have the maximum dry densities (i.e., 1691 kg/m^3^ and 1871 kg/m^3^) and moisture content of 2% (similar to the mean moisture content of mud dauber nests). The penetrometer resistances were compared to those of mud dauber nest samples as shown in Fig. 7. The statistical data of this comparison is summarized in Supplementary Table S4. As shown in Fig. 7, penetration resistances of mud dauber nests are comparable to the standard Proctor compacted samples. This implies that the vibratory compaction used by mud daubers (i.e., repetitive tapping produced by the front legs and mandibles) is effective to compact soils. Although mud daubers are lightweight, the achieved dry densities and penetration resistances of the nests are similar to those of the standard Proctor compacted soil samples that were compacted by a 24.5 N rammer dropping from a height of 305 mm for 75 times with a total compactive effort of 600 kJ/m^3^.

### SEM, EDS, and XRD analysis

The SEM images of the nest soil samples are shown in Supplementary Fig. S4a, b, c, and d. The SEM images show that the soil particles are densely packed, which is also evidenced by the limited pore space existing in the nest soils as shown in the SEM images. The clay particles serve as cementing agents that coated (see labels A1, A2, and A3 in Supplementary Fig. S4) and cemented silt and sand particles (see labels B1, B2, and B3 in Supplementary Fig. S4) and filled pore space between silt and sand particles (see labels C1 and C2 in Supplementary Fig. S4). Supplementary Fig. S4e shows the EDS spectrum of a nest soil sample. The elemental composition of nest soil includes carbon, oxygen, sodium, magnesium, aluminum, silicon, potassium, calcium, iron, and copper, which are common elements existing in the soil. The detected aluminum, sodium, potassium, and magnesium could be elements in clay minerals that were detected in the XRD analysis. Supplementary Fig. S5 shows the XRD spectra of nest soil samples. The XRD spectra are similar between nest samples from different locations. The detected minerals include quartz, albite, calcite, nontronite, and illite. Quartz is a primary mineral component in the nest soil, which largely exceeds the fractions of other minerals. This observation was also confirmed by Polidori et al.^15^ who reported that quartz is a primary mineral of mud dauber nest soils. Nontronite (in the group of smectite minerals) was commonly seen in all samples, while illite (i.e., hydrous mica) was only detected in the samples from locations C, D, and E. These two clay minerals can serve as cementing agents to coat and cement silt and sand particles and fill the pores as shown in the SEM images (see Supplementary Fig. S4a, b, c, and d), producing high soil density and improving the strength of the mud dauber nests. The role of clay minerals in cementing soil particles was also found in the soil nests constructed by hirundine species^22^.

## Discussions

Mud daubers can produce a repetitive tapping (i.e., vibratory compaction) to compact nest soil using their front legs and mandibles. The resulted dry densities and penetration resistances of the nests were comparable to those of the standard Proctor compacted soil samples. It is worth noting that a 24.5 N rammer was dropped from a height of 205 mm 75 times in the standard Proctor compaction tests, while a female mud dauber solitarily completes the nest construction within several hours to one day without using a high compacting mass. This demonstrates the high effectiveness of the repetitive tapping used by mud daubers for compacting soils.

Based on our observation, black and yellow mud daubers can modulate soil moisture content and control the collected soil in the plastic state (i.e., soil can be remolded and has relatively low strength like Play-Doh), allowing them to form the collected soil into soil balls and carry them to the nest location. After plastering and compacting soil on the nests, the nest soil was dried from the plastic state to the solid state (i.e., soil is hardened during drying and accompanied by increasing strength) due to moisture loss under ambient humidity and temperature conditions. This is confirmed by the low moisture contents of the mud dauber nests that range from 0.65 to 4.12% as shown in Fig. 3a. At particle scale, water menisci are discontinuous in soil pores at low moisture content, forming strong capillary cohesion between soil particles. The strong capillary cohesion results in high soil strength at the macro scale^25^.

As shown in Fig. 5a, the resulted grain size distributions of nest soils include the sand fraction of 22.5% to 45.5%, silt fraction of 44.9% to 68.8%, and clay fraction of 8% to 13.4%. The coefficients of uniformity ranged from 7.1 to 44.3. The coefficients of curvature ranged from 0.5 to 3.6. These two coefficients indicate that the mud dauber nest soils are well-graded (i.e., coefficient of uniformity larger than 6 and the coefficient of curvature between 1 and 4)^30^. The well-graded grain size distributions can improve particle packing with small particles (e.g., clay) filling in the pore space of large particles (e.g., silt and sand) (see Supplementary Fig. S4), reducing pore space and increasing soil density. The comparisons of grain size distributions between nest soils and control soils (Fig. 5b and c) also demonstrate that mud daubers select specific sizes of soil particles (i.e., sandy silt with trace of clay or silt with some sand and trace of clay, Fig. 5a) to build their nests, which is therefore independent of geographical locations.

Although clay fraction only ranged from 8% to 13.4% in the nest soils, clay can play a significant role in increasing nest soil strength and density by cementing silt and sand particles together and filling their pore space, which is confirmed by the SEM images (see Supplementary Fig. S4). At low moisture content, the water menisci are bounded between platy clay particles, creating strong capillary cohesion and leading to the high strength of the nest soil. Similarly, clay is also used as a cementing agent for improving the strength of the soil nests built by other animals, such as termite and hirundine species^21–23^.

The nest soils were classified as sandy silt with trace of clay (ML) or silt with some sand and trace of clay (ML), which is often regarded as silty loess soils (i.e., collapsible soil)^42,43^. Howayek et al.^43^ compiled grain size distributions of silty loess soils in the literature and proposed grain size distribution boundaries for silty loess soil. The grain size distributions of mud dauber nest soils were compared to the upper and lower boundaries of the silty loess as shown in Fig. 5a. The nest soils are not silty loess as nest soils have a higher sand fraction. The higher sand fraction can reduce the collapse potential of nest soil when wetting as sand particles can behave as load-bearing skeletons in the soil matrix, potentially improving the durability of mud dauber nests.

It was reported that termites use their salivary secretions to enhance soil cementation^23^ and erosion-resistance^24^. However, it is still unclear whether mud daubers use their salivary secretion to cement nest soil. Polidori et al.^15^ reported that mud daubers did not use saliva in the nest construction based on the low organic contents measured in the nest soil, which contradicts earlier studies in which mud daubers’ saliva was used to cohere soil particles^40,41^. In this study, we explored the effect of salivary secretions on the strength of mud dauber nests by performing standard Proctor compaction tests on the smashed nest soil followed by penetrometer tests. The hypothesis is that if salivary secretions were used to cement nest soil, the standard Proctor compacted soil samples with the same dry densities and moisture contents of mud dauber nests should have lower penetration resistances than mud dauber nests. This is because the standard Proctor compacted soil samples were produced by the nest soil that was smashed using a mortar and pestle, which eliminated the cementation contributed by salivary secretions. However, the penetration resistances of the standard Proctor compacted soil samples, which have similar densities and moisture contents as the mud dauber nests, matched the penetration resistances of mud dauber nests (Fig. 7). This comparison demonstrates that the salivary secretions do not significantly contribute to soil cementation and improve the strength of mud dauber nests.

This study showed that the high strengths and high densities of black and yellow mud dauber nests were attributed to (1) vibratory compaction performed by mud daubers, (2) high capillary cohesion in the nest soils due to low moisture content, (3) well-graded soil grain size distribution, and (4) clay minerals serving as cementing agents in the nest soils. These factors can inspire civil engineers to develop resilient and durable earthen constructed buildings and sustainable soil improvement techniques (e.g., bio-inspired vibratory compaction), which will be explored in future studies.

## Supplementary Information

Supplementary Information.

## Data Availability

The datasets generated during and/or analyzed during the current study are available from the corresponding author on reasonable request.
