# Supplementary material for: Investigating physical and mechanical properties of nest soils used by mud dauber wasps from a geotechnical engineering perspective
Source: Sci Rep. 2022 Feb 9;12:2192. doi: 10.1038/s41598-022-06162-2 (PMC8828900; doi:10.1038/s41598-022-06162-2)
Supplement: Supplementary file 1 — Supplementary Information. [file 41598_2022_6162_MOESM1_ESM.pdf]

Investigating Physical and Mechanical Properties of Nest Soils Used by Mud Dauber Wasps from a Geotechnical Engineering Perspective

Joon S. Park[1,+], Noura S. Saleh[1,+], Hai Lin[2,*], Hussein Alqrinawi[1,+], Nathan P. Lord[3,+]

[1] Graduate Student, Department of Civil and Environmental Engineering, Louisiana State University, Baton Rouge, LA 70803, USA.

[2] Assistant Professor, Department of Civil and Environmental Engineering, Louisiana State University, Baton Rouge, LA 70803, USA.

[3] Assistant Professor, Department of Entomology, Louisiana State University, Baton Rouge, LA 70803, USA.

[*] Corresponding author: hailin1@lsu.edu.

[+] Coauthors: jpar168@lsu.edu, nsaleh2@lsu.edu, halqri1@lsu.edu, nlord@lsu.edu.

SUPPLEMENTARY INFORMATION

Supplementary Figures

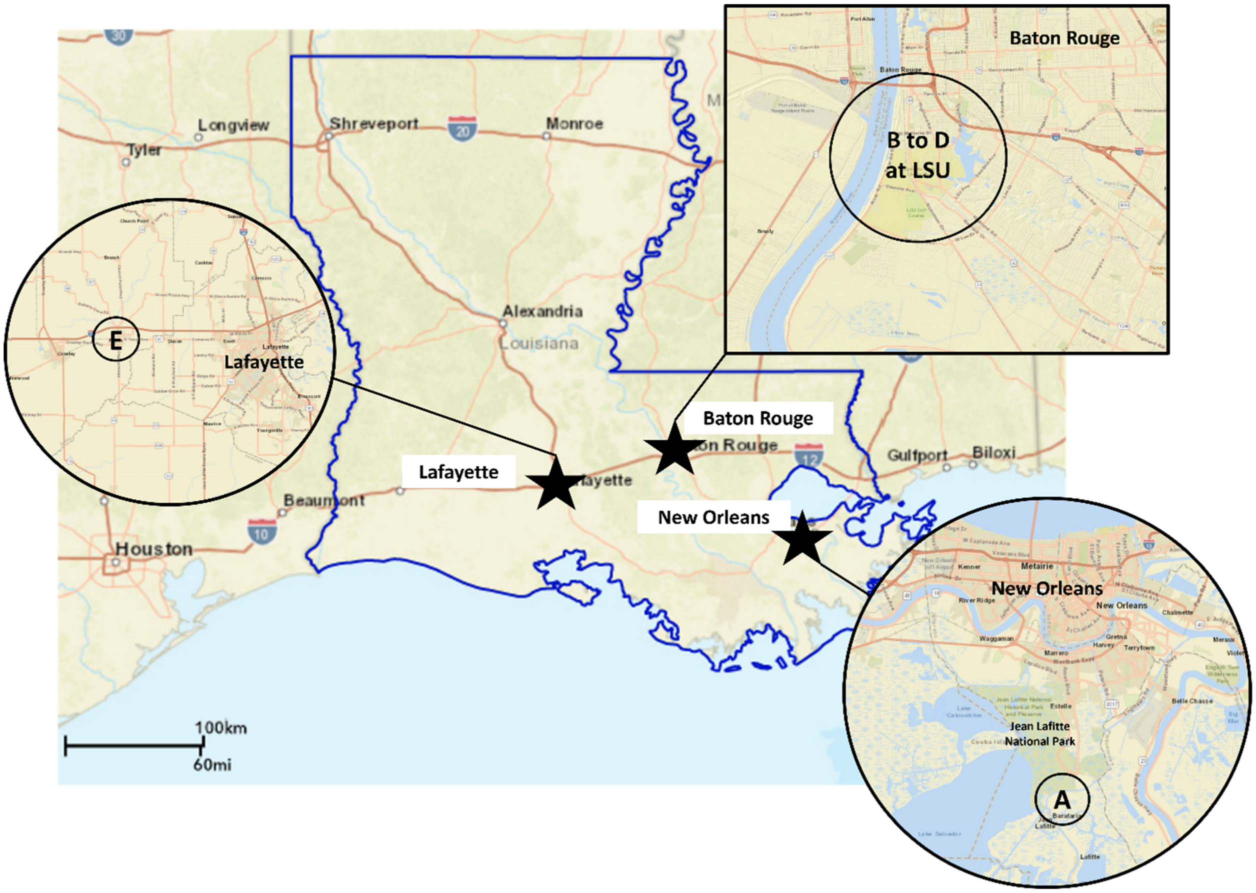

Figure S1. Sample collection map (created using ArcGIS Pro, https://www.esri.com, and Microsoft PowerPoint, https://www.microsoft.com).

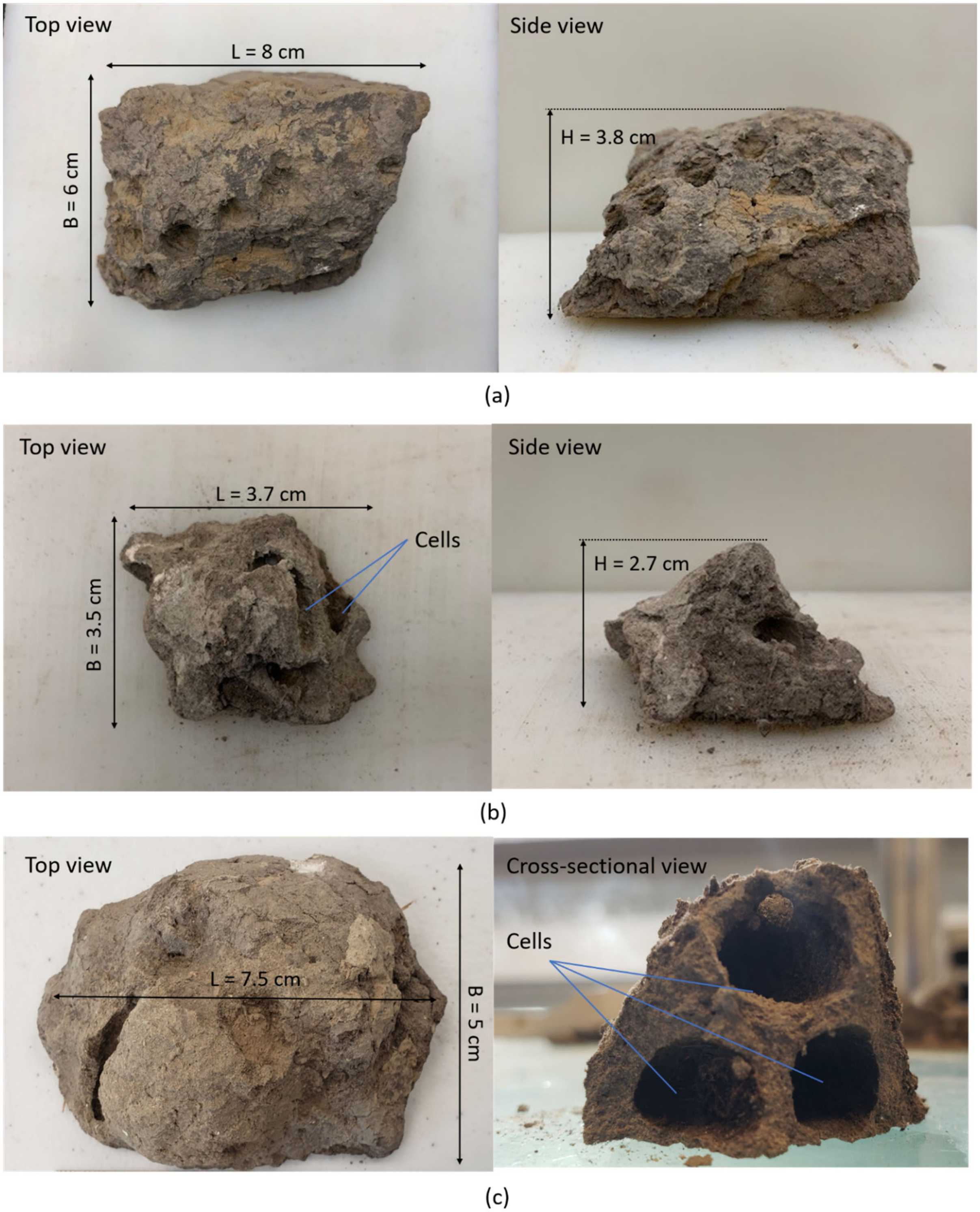

Figure S2. Characteristics of three collected mud dauber nests.

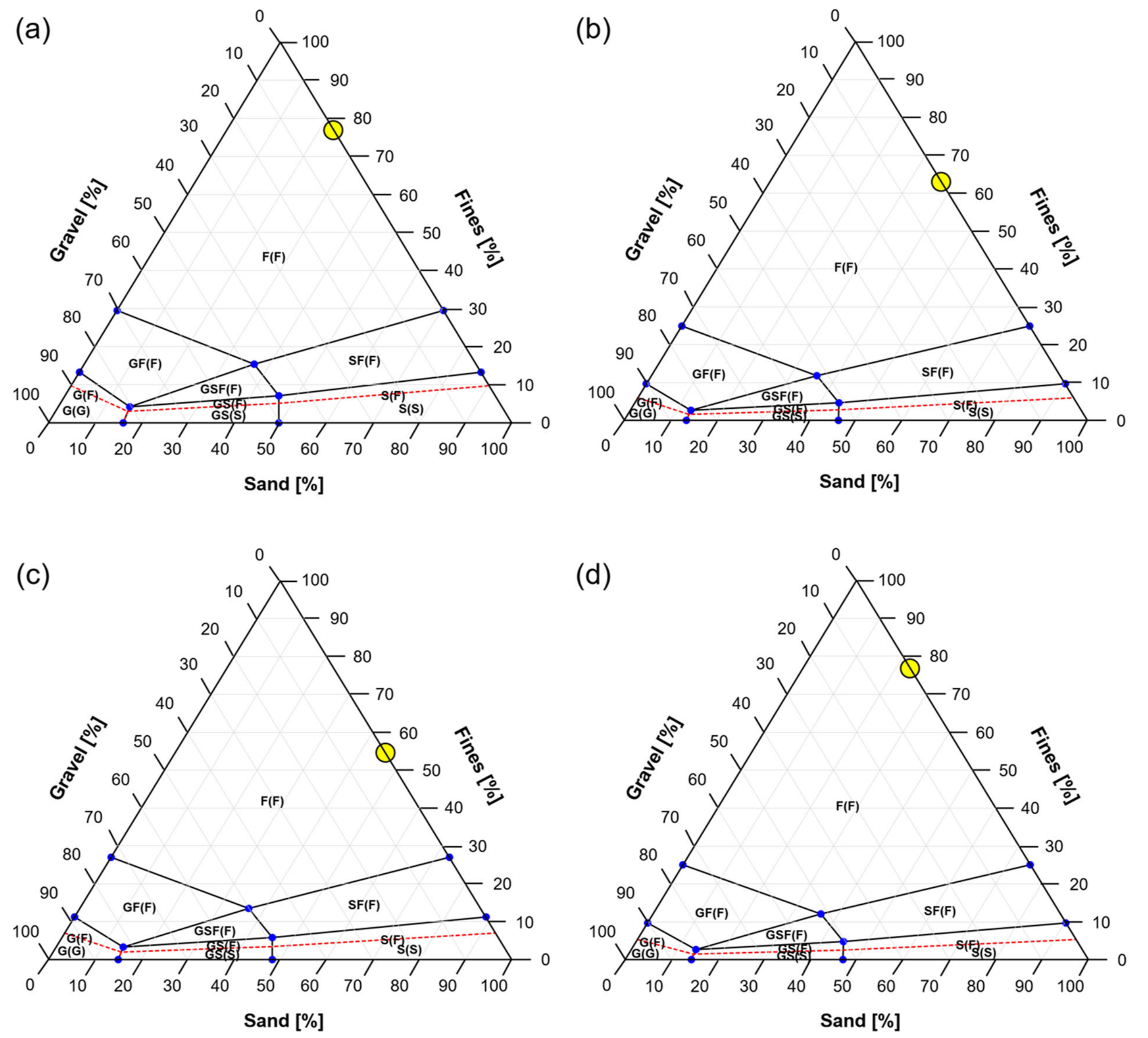

Figure S3. RSCS charts of the nest soil samples from (a) location A, (b) location B, (c) location C, and (d) location D.

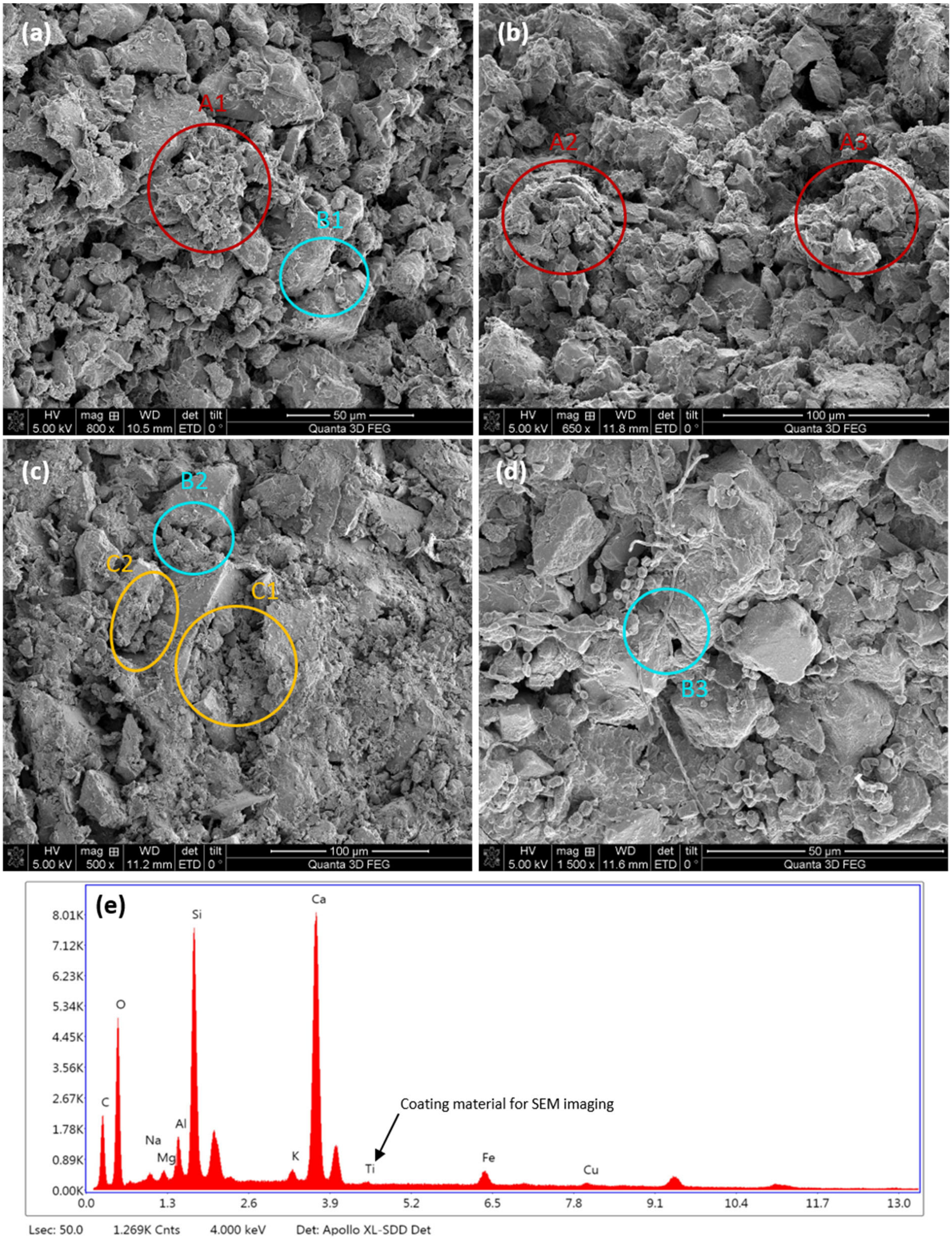

Figure S4. SEM images (a, b, c, and d) and EDS spectrum (e) of nest soils. A1-A3, clay coating silt and sand particles; B1-B3, clay cementing silt and sand particles; C1 and C2, clay filling pore space between silt and sand particles.

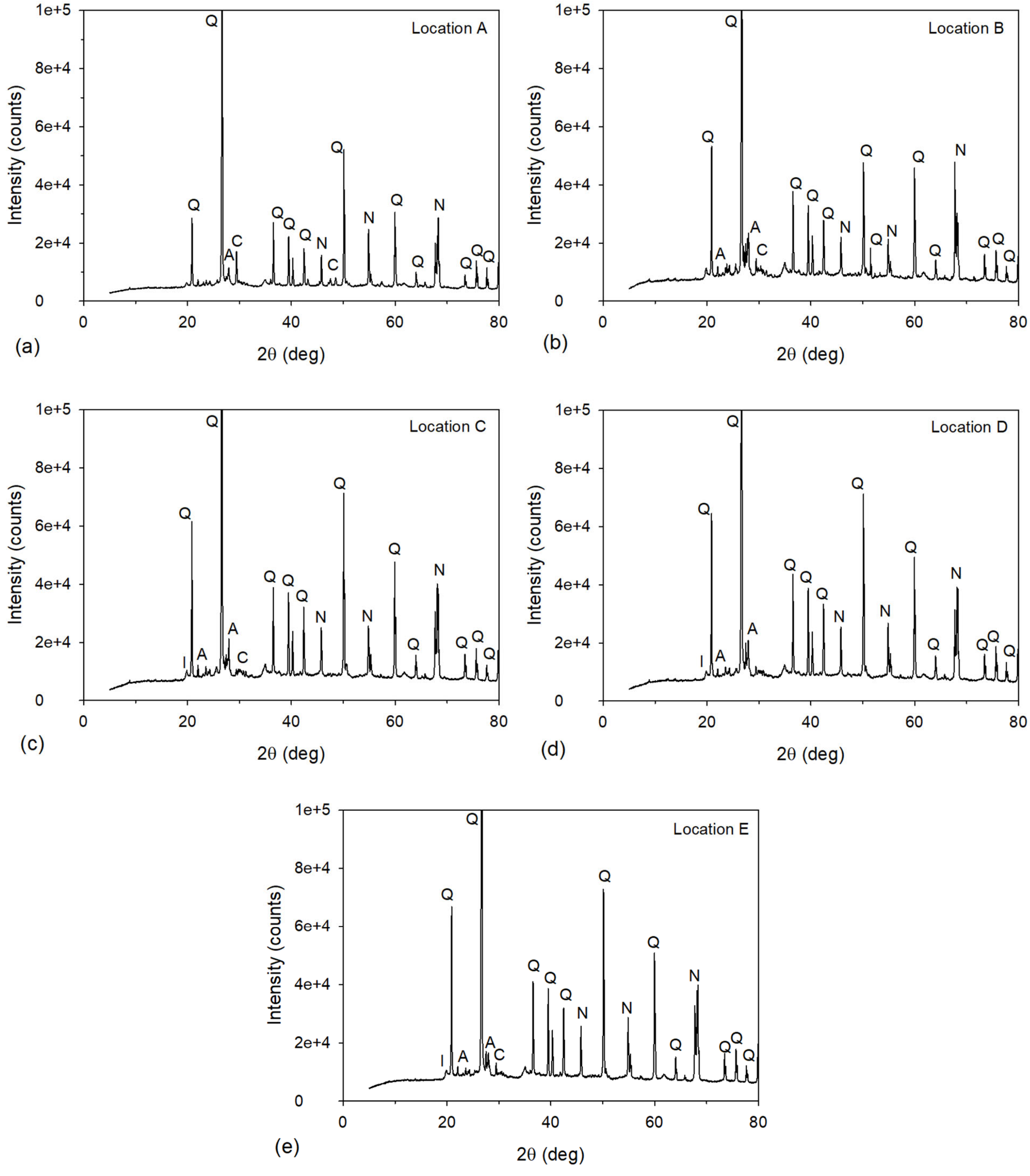

Figure S5. X-ray diffraction (XRD) spectra of the nest soil samples from (a) location A, (b) location B, (c) location C, (d) location D, and (e) location E. Q, quartz; A, albite; C, calcite; N, nontronite; and I, illite.

Supplementary Tables

Table S1. Statistical data of penetration resistances (kPa).

Locations	Number of penetrometer tests	Penetration resistances (kPa)			
		Mean	Median	Q1	Q3
All locations	711	466	408	268	585
A	614	447	397	263	568
B	14	371	370	185	464
C	23	703	636	383	931
D	25	474	407	313	617
E	35	670	586	442	899

Table S2. Physical properties of nest samples.

Physical properties	Locations	Number of tests	Mean	Median	Q1	Q3
Moisture content (%)	All locations	131	2.22	2.17	1.71	2.72
	A	94	2.39	2.35	1.89	2.94
	B	12	1.74	1.69	1.39	1.908
	C	10	1.77	1.73	1.44	2.21
	D	9	1.51	1.40	1.29	1.74
	E	6	2.26	2.18	2.02	2.48
Organic content (%)	All locations	131	5.16	4.78	3.33	6.70
	A	94	5.79	5.46	3.73	7.37
	B	12	3.78	3.44	2.53	5.07
	C	10	3.51	3.24	2.48	4.32
	D	9	3.15	2.94	2.22	3.84
	E	6	5.92	5.78	4.56	7.14
Dry density, ρ_d (kg/m^3)	All locations	236	1587	1588	1483	1699
	A	153	1559	1552	1458	1652
	B	24	1591	1648	1474	1701
	C	21	1694	1755	1544	1804
	D	23	1653	1658	1582	1767
	E	15	1556	1568	1492	1639
Void ratio, e	All locations	236	0.65	0.64	0.53	0.75
	A	153	0.64	0.63	0.53	0.74
	B	24	0.65	0.58	0.53	0.76
	C	21	0.55	0.48	0.44	0.68
	D	23	0.59	0.57	0.47	0.64
	E	15	0.65	0.65	0.58	0.74

Table S3. Input parameters for RSCS analysis.

Nest location	A	B	C	D
[a]Sand fraction, F_s (%)	22.5	37	45.5	23.2
[b]Fine fraction, F_F (%)	77.5	63	54.5	76.8
C_u [c]	1.95	3.1	2.43	2.75
R [d]	0.7	0.7	0.7	0.7
LL [e] (%)	46.06	N/A	N/A	33.8

[a]Between grain sizes of 75 and 4000 μm

[b]Grain size less than 75 μm

[c]Coefficient of uniformity of sand fraction

[d]Roundness of sand fraction

[e]Liquid limit of fine fraction

Note: Calculation equations are provided in Park and Santamarina[29].

Table S4. Comparison of measured penetration resistances (kPa) between the mud dauber nests and laboratory compacted samples.

Compaction methods	Number of tests	Mean	Median	Q1	Q3
Mud dauber	711	466	408	268	585
Standard Proctor	444	456	383	281	539
Modified Proctor	379	606	482	346	738
